# Increased plasma VEGF levels following ischemic preconditioning are associated with downregulation of miRNA-762 and miR-3072-5p

**DOI:** 10.1038/srep36758

**Published:** 2016-12-01

**Authors:** Koji Ueno, Makoto Samura, Tamami Nakamura, Yuya Tanaka, Yuriko Takeuchi, Daichi Kawamura, Masaya Takahashi, Tohru Hosoyama, Noriyasu Morikage, Kimikazu Hamano

**Affiliations:** 1 Department of Surgery and Clinical Sciences, Yamaguchi University Graduate School of Medicine, Ube, Japan; 2 Center for Regenerative Medicine, Yamaguchi University Graduate School of Medicine, Ube, Japan

## Abstract

Ischemic preconditioning (IPC) has protective effects against ischemia-perfusion injury of organs. In the present study, we investigated the associated mechanisms after performing remote IPC (rIPC) of lower limbs by clamping abdominal aorta in mice. Subsequent experiments showed decreased damage and paralysis of lower limbs following spinal cord injury (SCI). Concomitantly, plasma vascular endothelial growth factor (VEGF) levels were increased 24 h after rIPC compared with those in sham-operated animals. In subsequent microRNA analyses, thirteen microRNAs were downregulated in exosomes 24 h after rIPC. Further studies of femoral CD34-positive bone marrow (BM) cells confirmed downregulation of these seven microRNAs 24 h after rIPC compared with those in sham-operated controls. Subsequent algorithm-based database searches suggested that two of the seven microRNAs bind to the 3′ UTR of VEGF mRNA, and following transfection into CD34-positive BM cells, anti-miR-762, and anti-miR-3072-5p inhibitors led to increased VEGF concentrations. The present data suggest that rIPC transiently increases plasma VEGF levels by downregulating miR-762 and miR-3072-5p in CD34-positive BM cells, leading to protection against organ ischemia.

Previous studies suggest that ischemic preconditioning (IPC) induces biological defense reactions that protect organs^1^, and myocardial infarction was limited following repetition of brief ischemia and reperfusion before the induction of myocardial infarction by left anterior descending artery (LAD)-ligation in dogs^2^,^3^. In agreement, IPC was protective of the liver, spinal cord, and kidney tissues^4^,^5^,^6^, and remote IPC (rIPC) was protective of distal organs^7^. Effective organ protection by IPC has been described as biphasic^8^, and the ensuing anti-apoptotic effects have been associated with endogenous factors such as nitric oxide and adenosine^9^ and with increased peripheral mobilization of bone marrow cells^10^. However, the mechanisms underlying effective organ protection by IPC remain poorly understood.

Although less than 2% of the total human genome sequence comprises protein-coding genes, 90% of genomic sequences are transcribed, and large-scale genome analyses have demonstrated that non-coding RNAs account for the majority of these^11^. Although percentages of non-coding RNAs differ between species, higher species have demonstrably greater proportions of non-coding RNAs among total RNAs than lower species^12^. MicroRNAs bind three prime untranslated regions (3′ UTR) of target gene mRNAs (messenger RNAs) and inhibit translation or induce mRNA cleavage^13^. In addition, microRNAs are secreted in exosomes that regulate protein translation following incorporation into cells of remote tissues. Accordingly, extracellular microRNAs (exRNAs) were reportedly associated with cell–cell transmission functions^14^.

Various spinal cord protection measures are performed during surgery. However, lack of blood flow can lead to blockage of spinal cord arteries during endovascular repair following thoracic aortic aneurysms. The resulting spinal cord ischemia (SCI) reportedly occurs in 1–7.5% of thoracic stent graft cases and causes temporary or permanent paraplegia^15^,^16^,^17^,^18^,^19^, warranting assessments of strategies that avoid SCI during vascular surgery and reduce the occurrence of paraplegia following thoracic stent graft procedures. Herein, we examined the utility of late phase rIPC following SCI and show that rIPC increases plasma VEGF concentrations and reduces the severity of paraplegia following SCI. After validating microarray data for exosome microRNAs, we identified microRNAs that are downregulated by rIPC. Specifically, Target Scan algorithms showed that miR-762 and miR-3072-5p target the 3′ UTR of VEGF mRNA and were downregulated after rIPC. Accordingly, IPC-induced expression levels of these microRNAs in CD34-positive BM cells from femurs were lower than those from sham-operated controls. In agreement, VEGF secretions were increased in CD34-positive BM cells following treatment with anti-miR-762 and anti-miR-3072-5p inhibitors. Taken together, the present data indicate that effective organ protection by rIPC reflects increased plasma VEGF concentrations following downregulation of miR-762 and miR-3072-5p in CD34-positive BM cells.

## Results

### Effects of organ protection and increases in plasma VEGF following rIPC

**Figure 1:**
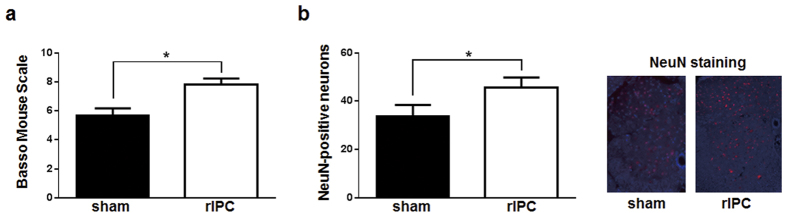
Protective effects of rIPC against paraplegia following spinal cord ischemia. (**a**) Evaluation of paraplegia using the Basso Mouse Scale; Remote ischemic preconditioning (rIPC) was performed by clamping the abdominal aorta. At 24 h after rIPC, SCI was induced by clamping the descending aortic cross. Paraplegia was then evaluated at 3 days after SCI using the Basso Mouse Scale. Each group included six mice. (**b**) Immunostaining against neural cell marker NeuN; third lumber spinal cords were immunostained using an anti-NeuN antibody on the day of Basso Mouse Scale assessments. Presented images show typical immunostaining against NeuN.

**Figure 2:**
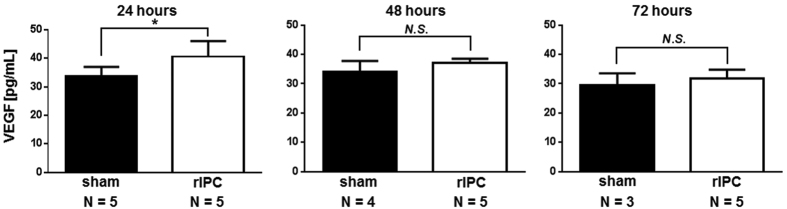
Comparison of vascular endothelial growth factor concentrations in plasma after rIPC. Vascular endothelial growth factor (VEGF) in plasma was measured using ELISA 24, 48, and 72 h after rIPC; mice numbers, N.

To evaluate the organ-protective effects of rIPC, rIPC or sham operations were performed 24 h before SCI and lower limb paralysis was quantified after 3 days. Basso Mouse Scale scores were significantly higher in the rIPC-treated group than in the sham group (Fig. 1a). Moreover, in immunostaining analyses of neurons in spinal cord tissues (Fig. 1b), significantly greater numbers of NeuN-positive neurons were observed in L3 vertebra from rIPC-treated mice than in those from sham treated mice (Fig. 1b). In subsequent experiments, plasma VEGF levels were determined using enzyme-linked immunosorbent assay (ELISA) 24, 48, and 72 h after rIPC. These experiments showed significantly higher plasma VEGF levels in the rIPC-treated group than in the sham group 24 h after rIPC, although VEGF concentrations did not differ between treatment groups 48 and 72 h after IPC (Fig. 2).

### Expression of exosome-encapsulated microRNAs in CD34-positive BM cells after rIPC

**Figure 3:**
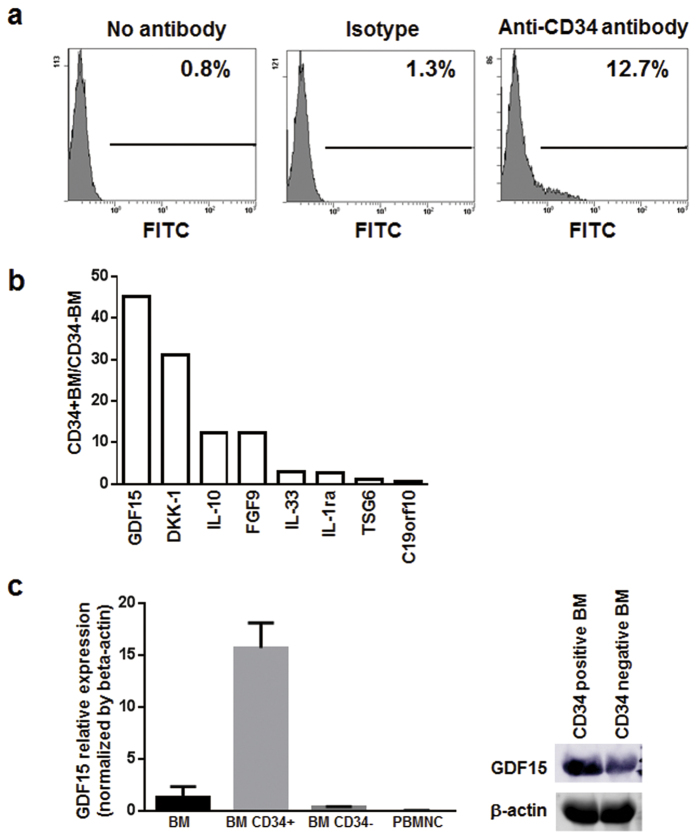
Characteristics of CD34-positive bone marrow cells. (**a**) Proportions of CD34-positive bone marrow (BM) cells were calculated using a FC500 flow cytometer, and (**b**) mRNA expression levels were compared between CD34-positive and CD34-negative BM cells using qPCR. ACTB was used as an endogenous control. Expression levels in CD34-positive BM cells are expressed relative to those in CD34-negative BM cells. (**c**) GDF15 expression levels were analyzed using qPCR with ACTB as an endogenous control, and are presented relative to those in BM cells.

**Figure 4:**
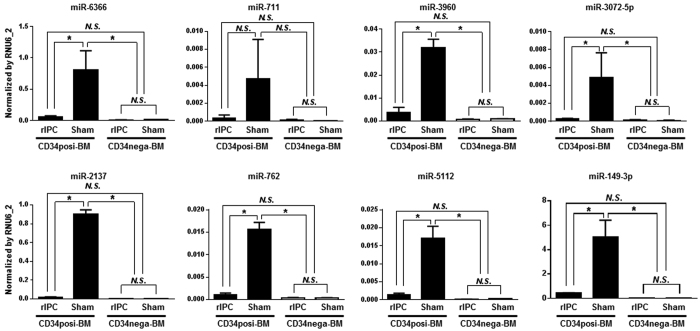
Changes in microRNA expression in bone marrow cells after rIPC. Expression levels of eight microRNAs were determined using qPCR in CD34-positive and CD34-negative BM cells after rIPC using RNU6_2 as an endogenous control. Expression data are presented relative to that of RNU6_2.

Changes in expression levels of exosomal microRNAs were determined following extraction from plasma 24 h after rIPC or sham treatments, and microRNA microarrays were performed. In exomes from rIPC treated animals, thirteen microRNAs were expressed at ≤0.3-fold those in sham treated animals (Table 1), and eighteen microRNAs were expressed in rIPC-treated animals at more than double their expression in the sham group (Table 2). Subsequent flow cytometry analyses showed that CD34-positive BM cells comprised about 11% of BM cells from femurs (Fig. 3a). Previously, it was reported that CD34-positive BM cells were mobilized in peripheral tissues 24 h after rIPC in mice 10. Thus, we compared mRNA expression of ischemia-reperfusion protective factors between CD34-positive and CD34-negative BM cells using qPCR (Fig. 3b). These experiments showed 15-fold higher expression of GDF15 mRNA in CD34-positive than in CD34-negative BM cells and significantly higher expression levels than in peripheral blood mononuclear cells (PBMNC) (Fig. 3c). In agreement, GDF15 protein levels were higher in CD34-positive than in CD34-negative BM cells (Fig. 3c). Thus, to further investigate relationships between increased plasma VEGF levels (Fig. 2) and the thirteen microRNAs (Table 1) that were downregulated following rIPC, expression levels of miR-6366, miR-711, miR-3960, miR-3072-5p, miR-2137, miR-762, miR-5112, and miR-149-3p were investigated in BM cells (Fig. 4). Although no differences in expression were identified between CD34-negative BM cells from rIPC and sham treated animals, seven microRNAs were downregulated in CD34-positive BM cells from IPC-treated mice.

### Database searches of microRNAs that increase VEGF expression after IPC

**Figure 5:**
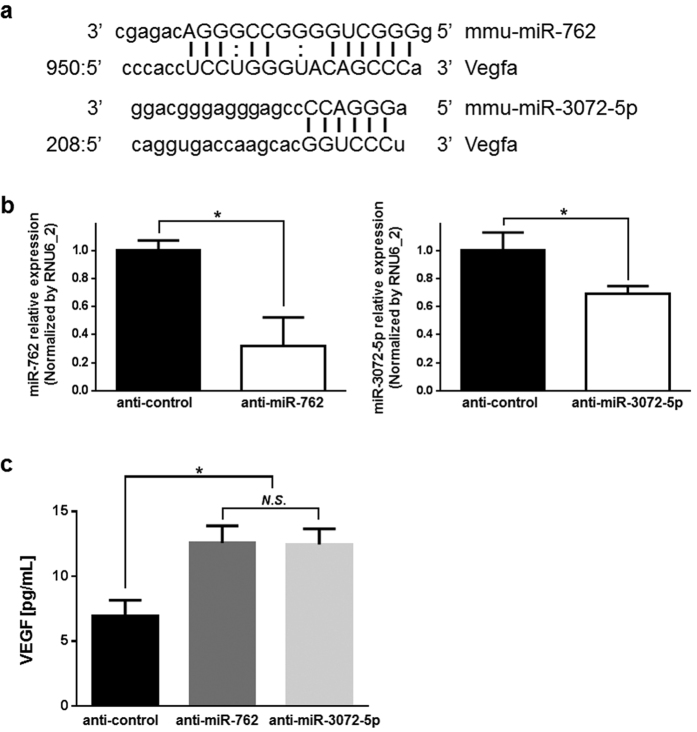
miR-762 and miR-3072-5p target VEGF. (**a**) VEGF 3′ UTR sequence and complementary miR-762 and miR-3072-5p-binding sequences. (**b**) Knockdown of microRNA expression after transfection with anti-miR-762 and anti-miR-3072-5p inhibitors. MicroRNA was extracted 24 h after transfection and microRNA expression levels were confirmed using qPCR with RNU6_2 as an endogenous control. Expression levels were presented relative to those in CD34-positive BM cells that were transfected with anti-control inhibitor. (**c**) VEGF secretion in CD34-positive BM cells 24 h after transfection. Anti-microRNA inhibitors were transfected into CD34-positive BM cells and VEGF concentrations in media were measured using ELISA 24 h after transfection.

To investigate mechanisms that lead to increases in plasma VEGF concentrations after rIPC, we searched for putative relationships between VEGF and the seven downregulated microRNAs using Target Scan algorithms (www.microRNA.org). VEGF mRNA has 3′ UTR sequences that are potentially complimentary with miR-762, and miR-3072-5p (Fig. 5a). Thus, to determine whether downregulation of miR-762 and miR-3072-5p leads to increased VEGF expression, expression levels of microRNA were confirmed using qPCR 24 h after transfection with anti-miR-762 and anti-miR-3072-5p inhibitors. MiRNA-762 and miR-3072-5p expression levels were downregulated in CD34-positive BM cells after transfection (Fig. 5b). Moreover, VEGF secretion levels were concomitantly doubled in both anti-miR-762 and anti-miR-3072-5p transfected CD34-positive BM cells compared with control transfected cells (Fig. 5c). To ensure that miR-762 and miR-3072-5p bind to the 3′ UTR of VEGF mRNA, their mimics were transfected into CD34-positive BM cells, and the concentrations of VEGF were measured with ELISA. Both miR-762 and miR-3072-5p mimics inhibited VEGF secretion in CD34-positive BM cells compared with control transfected cells (Supplementary Figure S1).

## Discussion

IPC has known spinal cord protective effects when administered prior to ischemia-reperfusion injury^5^,^20^,^21^,^22^,^23^,^24^. Whereas IPC is performed directly (dIPC) and remotely, rIPC has demonstrated beneficial effects on spinal cord neurons following ischemia^25^,^26^,^27^. IPC-induced organ protective effects have also been described as biphasic, with the early phase within the first 3 h and the late phase between 12 h and 4 days^8^. Although spinal cord protection by rIPC reportedly proceeds during the early phase, our data suggest that the late phase effects of rIPC might offer protection of the spinal cord from ischemic injury.

In a previous study, dIPC induced VEGF mRNA during myocardial infarction between 3 and 12 h after ligation of the left anterior descending coronary artery (LAD)^28^, and dIPC in the kidney reportedly increased VEGF mRNA in renal tissues between 1 and 6 h, and increased renal VEGF protein levels at 24 h^29^. These studies both show that VEGF mRNA expression returns to baseline levels 24 h after IPC. In agreement, we show high plasma VEGF protein contents 24 h after rIPC and restored levels at 48 h. In consideration of reported neuron protective effects of VEGF in an *in vitro* ischemic model^30^, the present and previous *in vivo* data indicate that rIPC induced VEGF is protective against spinal cord ischemia.

Beneficial effects of anti-inflammatory agents have been reported for spinal cord injury^31^,^32^, and these effects are in accordance with increased mRNA expression levels of anti-inflammatory, angiogenic, and ischemia resistance genes in CD34-positive BM cells than in CD34-negative BM cells^33^,^34^,^35^,^36^,^37^,^38^,^39^,^40^,^41^. In particular, GDF15, Dkk1, IL33, and FGF9 mRNA expression levels were more than 10-fold higher in CD34-positive than in CD34-negative BM cells. Moreover, after 72-h culture on fibronectin, human CD34-positive endothelial progenitor cells from umbilical cord blood expressed anti-inflammatory GDF15 at high levels^42^,^43^. Hence, GDF15 may be secreted from CD34-positive cells that have been mobilized in peripheral blood following IPC, explaining the protective effects against spinal cord ischemia.

In an early study of exosome microRNAs in blood and the protective effects of rIPC against ischemia, miR-144 expression levels increased in mouse hearts and plasma after rIPC and reduced myocardial ischemia-reperfusion injury^44^. Similarly, the present comprehensive *in vivo* microarray analyses (Mouse_miRNA_V19) of exosomes after rIPC, identified numerous differentially expressed microRNAs were validated using qPCR except miR-3067-3p, miR-2861, and miR-5126 because primer designs were hampered by GC-rich sequences of these microRNAs. Downregulated microRNAs that target the 3′ UTR of VEGF mRNA were investigated as causes of increased plasma VEGF concentrations 24 h after IPC, and Target Scan algorithms (www.microRNA.org) predicted that miR-762 and miR-3072-5p bind the 3′ UTR of VEGF mRNA. Increased proportions of CD34-positive BM cells have previously been shown in blood^10^, suggesting involvement in VEGF production after rIPC. Accordingly, the present data show that anti-miR-762 and anti-miR-3072-5p inhibitors increased VEGF secretion levels in CD34-positive BM cells 24 h after transfection. Moreover, the respective miRNAs miR-762 and miR-3072-5p reportedly target the 3′ UTR of VEGF mRNA. Thus, increased plasma VEGF concentrations are likely associated with downregulation of mR-762 and miR-3072-5p 24 h after rIPC.

In conclusion, this is the first study to show that rIPC has protective effects against spinal cord injury following ischemia. In addition, the present mechanistic data indicate that CD34-positive BM cells produce VEGF by downregulating miR-762 and miR-3072-5p, and that VEGF protects spinal cord neurons against ischemia. Although additional studies are required to define the mechanisms involved in the relationship between VEGF production and neuron protection, rIPC may be a useful method for decreasing the occurrence of spinal cord ischemia and limb paralysis following thoracic stent graft procedures.

## Materials and Methods

### Animals

Male C57BL/6 mice were purchased from Japan SLC, Inc. (Shizuoka, Japan). All animal procedures were approved by the Institutional Animal Care and Use Committee of Yamaguchi University (#31-087) and all methods were conducted in accordance with the approved guidelines.

### Ischemic preconditioning

Mice were anesthetized by inhalation of 1.5% isoflurane. Ischemic preconditioning was performed in abdominal aorta using three cycles of 5-min clamping followed by 5 min of reperfusion. Sham laparotomy was performed in control animals 10.

### Spinal cord ischemia

Mice were anesthetized by inhalation of 1.5% isoflurane and were then intubated using a 20-gauge intravenous catheter. SCI was then induced using descending aortic cross clamping at 10 min^45^,^46^.

### Effects of ischemic preconditioning on spinal cord ischemia

SCI was induced 24 h after ischemic preconditioning or sham laparotomy. At 3 days after induction of SCI, the effects of ischemic preconditioning were evaluated using the Basso Mouse Scale for Locomotion^47^.

### Histological analysis

Spinal cords were collected from animals after reflux with saline and were fixed in 10% Formalin Neutral Buffer Solution (Wako, Osaka, Japan) with shaking for 3 days at room temperature. After transferring to water to remove extra formalin, spinal cords were degreased in ethanol overnight at room temperature, and were again transferred to water to remove extra ethanol. Subsequently, spinal cords were soaked in double diluted K-CX (Falma, Tokyo, Japan) for 3 days at 4 °C, were transferred to water to remove K-CX, and were then neutralized with 5% sodium sulfate overnight at room temperature. Spinal cords were then washed five times with water for 1 h at room temperature. Third lumber spinal cords (L3) were then excised and embedded in paraffin blocks for immunohistochemical analyses using an anti-NeuN monoclonal antibody (#MAB377, Merck Millipore, Darmstadt, Germany) and the anti-mouse IgG (H + L) secondary antibody Alexa Fluor^®^ 594 conjugate (A-11005, Thermo Fisher Scientific, Waltham, Massachusetts, USA).

### VEGF measurement

Blood samples were collected with heparin at 1, 2, and 3 days after ischemic preconditioning and plasma VEGF analyses were performed in supernatants after centrifugation at 3,000 rpm for 10 min. VEGF analyses were performed in cultured media that was collected 24 h after transfection and centrifuged at 2,000 rpm for 3 min. VEGF concentrations in plasma and cell culture supernatants were measured using mouse ELISA kits (R&D Systems, Inc., Minneapolis, Minnesota, USA) according to the manufacturer’s instructions.

### MicroRNA isolation from exosomes and microarray analyses

Plasma was collected with EDTA 24 h after ischemic preconditioning or sham laparotomy. MicroRNA was extracted from plasma exosomes using Total Exosome Isolation Kits (Thermo Fisher Scientific) and Total Exome RNA and Protein Isolation Kits (Thermo Fisher Scientific). MicroRNA microarray analyses were performed using 3D-Gene^®^ miRNA Oligo chips from Toray Industries Inc. (Kamakura, Kanagawa, Japan).

### Flow cytometry and isolation of CD34-positive BM cells

BM cells were collected from femurs by flushing with PBS using a syringe, filtering through a 40-μm cell strainer, and treating with ammonium chloride for hemolysis. Bone marrow cells were incubated with FITC Rat anti-Mouse CD34 (#553733, BD Biosciences, San Jose, California, USA) or FITC Rat IgG2a κ Isotype Control (#553929, BD Biosciences) on ice for 60 min. Analyses were then performed using a Cytomics FC500 with FC500 CXP Cytometer software (Beckman Coulter Co., Miami, FL, USA). CD34-positive and CD34–negative bone marrow cells were separated using a MACS Cell Separation instrument (Miltenyi Biotec, Bergisch Gladbach, Germany) with Purified Rat Anti-Mouse CD34 (#553731,BD Biosciences) and Anti-Rat IgG MicroBeads (Miltenyi Biotec).

### Quantitative PCR

Peripheral blood mononuclear cells (PBMNC) were isolated from mouse peripheral blood samples using Lympholyte^®^-M (CedarLane Laboratories Ltd., Hornsby, Ontario, Canada). Total RNA and microRNAs were extracted from CD34-positive and CD34-negative BM cells and PBMNC using RNeasy Mini Kits and miRNeasy Mini Kits (Qiagen, Hilden, Germany). Extracted total RNA was then reverse-transcribed into single-stranded cDNA using PrimeScript™ RT reagent Kits (Perfect Real Time; TaKaRa Bio Inc., Kusatsu, Shiga, Japan). Real-time PCR was performed using first-strand cDNA with SYBR^®^ Select Master Mix (Thermo Fisher Scientific) and the following primers: ACTB forward primer, 5′-GCTCCTCCTGAGCGCAAG-3′; ACTB reverse primer, 5′-CATCTGCTGGAAGGTGGACA-3′; GDF15 forward primer, 5′-AGAGGACTCGAACTCAGAACCAA-3′; GDF15 reverse primer, 5′-CCCCAATCTCACCTCTGGAC-3′; DKK-1 forward primer, 5′-TGGAATATGCATGCCCTCTG-3′; DKK-1 reverse primer, 5′-GCGGCGTTGTGGTCATA-3′; IL-10 forward primer, 5′-CAGCCAGGTGAAGACTTTCTTTC-3′; IL-10 reverse primer, 5′-CAACCCAAGTAACCCTTAAAGTCC-3′; FGF9 forward primer, 5′-TGCAGGACTGGATTTCATTTAGAG-3′; FGF9 reverse primer, 5′-AAGCGGCTGTGGTCTTTCC-3′; IL-33 forward primer, 5′-AAAATCGGGTACCAAGCATGAA-3′; IL-33 reverse primer, 5′-TGTGTCAACAGACGCAGCAA-3′; IL-1ra forward primer, 5′-CCTTCAGAATCTGGGATACTAACCA-3′; IL-1ra reverse primer, 5′-CGTGGATGCCCAAGAACA-3′; TSG6 forward primer, 5′-GTCCACGGCTTTGTAGGAAGATA-3′; TSG6 reverse primer, 5′-GACGGATGCATCACTCAGAAAC-3′; C19orf10 forward primer, 5′-CCAACGAGCAATGGCAGA-3′; and C19orf10 reverse primer, 5′-gcctccagatggtacaggtaaag-3. Quantitative PCR was performed using a StepOnePlus Real-Time PCR System (Thermo Fisher Scientific) and polymerase chain reactions were performed at 50 °C for 2 min followed by 95 °C for 2 min and 40 cycles of 95 °C for 3 s and 60 °C for 30 s. All reactions were performed in 10-μl reaction volumes in triplicate and mRNA expression levels were determined using the 2^−ΔCT^ method. Extracted microRNAs were reverse-transcribed into single-stranded cDNA using a miScript II RT Kit (Qiagen), and real-time PCR was performed using first strand cDNA with miScript SYBR Green PCR Kit (Qiagen) and the following miScript Primer Assays (Qiagen): RNU6_2 (MS00033740), miR-149-3p (MS00069222), miR-711 (MS00002975), miR-762 (MS00016443), miR-2137 (MS00021917), miR-3072-5p (MS00021938), miR-3960 (MS00042777), miR-5112 (MS00043008), and miR-6366 (MS00064917). Quantitative PCR was performed using a StepOnePlus instrument (Thermo Fisher Scientific) at 50 °C for 2 min followed by 95 °C for 15 min and 40 cycles at 94 °C for 15 s, 55 °C for 30, and 70 °C for 30 s. All reactions were performed in 10-μl reaction volumes in triplicate and microRNA expression levels were normalized to those of the internal control.

### Western blot analysis

Cells were re-suspended in cold buffer containing 10-mM Tris-HCl (pH 7.4), 0.5-mM EGTA, 0.5-mM EDTA, 1% Triton X-100, and cOmplete™ Mini Protease Inhibitor Cocktail (Sigma, St Louis, MO, USA) and were incubated for 30 min on ice and then centrifuged at 12 000 *g* for 20 min at 4 °C. Supernatants containing total protein were collected and subjected to Western blotting using rabbit anti-GDF15/MIC-polyclonal antibody (1:500 dilution, # bs-3818R, Bioss, Woburn, Massachusetts, USA), anti-rabbit IgG HRP-conjugated secondary antibody (1:5000 dilution, #P0448, Dako, Glostrup, Denmark), and anti-β-actin HRP-conjugated antibody (1:2000 dilution #NB600-532H, Novus Biologicals, Littleton, Colorado, USA). Extracted proteins were visualized using Amersham ECL Prime Western Blotting Detection Reagent (#RPN2232, GE Healthcare Life Sciences, Little Chalfont, United Kingdom).

### Transfection

For microRNA knockdown experiment, negative control, mmu-miR-762, and mmu-miR-3072-5p miScript miRNA inhibitors (Qiagen) were transfected into cells using HiPerFect Transfection Reagent (Qiagen). Subsequently, 8 × 10^4^ CD34-positive BM cells were seeded in 250 μl of medium into 48-well tissue culture plates, and 4.5 μl of 20-μM inhibitor and 4.5 μl of HiPerFect Transfection Reagent were added to 50 μl of Opti-MEM^®^ I Reduced Serum Media (Thermo Fisher Scientific), were vortexed and centrifuged, and were then applied to cells after incubation for 15 min. For microRNA mimic transfection, control, mmu-miR-762, and mmu-miR-3072-5p mimics (GeneDesign, Inc., Osaka, Japan) were transfected into cells as a double strand RNA using HiPerFect Transfection Reagent (Qiagen) and the following RNA sequence: control sense strand, 5′-AAAUCGCUGAUUUGUGUAGUC-3′; control antisense strand, 5′-GACUACACAAAUCAGCGAUUU-3′; mmu-miR-762 sense strand, 5′-GGGGCUGGGGCCGGGACAGAGC-3′; mmu-miR-762 antisense strand, 5′-GCUCUGUCCCGGCCCCAGCCCC-3′; mmu-miR-3072-5p sense strand, 5′-AGGGACCCCGAGGGAGGGCAGG-3′; mmu-miR-3072-5p antisense strand, 5′-CCUGCCCUCCCUCGGGGUCCCU-3′. Subsequently, 1.4 × 10^5^ CD34-positive BM cells were seeded in 250 μl of medium into 48-well tissue culture plates, and 3 μl of 100-μM mimic and 4.5 μl of HiPerFect Transfection Reagent were added to 50 μl of Opti-MEM^®^ I Reduced Serum Media (Thermo Fisher Scientific), were vortexed and centrifuged, and were then applied to cells after incubation for 15 min.

### Statistical analysis

All statistical analyses were performed using GraphPad Prism 6 software (GraphPad Software, San Diego, CA, USA). Differences were considered significant when P < 0.05.

## Additional Information

**How to cite this article**: Ueno, K. *et al*. Increased plasma VEGF levels following ischemic preconditioning are associated with downregulation of miRNA-762 and miR-3072-5p. *Sci. Rep.* **6**, 36758; doi: 10.1038/srep36758 (2016).

**Publisher's note:** Springer Nature remains neutral with regard to jurisdictional claims in published maps and institutional affiliations.

## Supplementary Material

Supplementary Information

## Figures and Tables

**Table 1:** Downregulated exosome microRNAs 24 h after rIPC.

microRNAs	Sham	rIPC	Fold change
mmu-miR-3067-3p	433	33	0.08
mmu-miR-6366	198	21	0.11
mmu-miR-2861	836	90	0.11
mmu-miR-711	412	53	0.13
mmu-miR-5126	1447	196	0.14
mmu-miR-3960	796	109	0.14
mmu-miR-3072-5p	281	42	0.15
mmu-miR-2137	865	135	0.16
mmu-miR-762	319	62	0.19
mmu-miR-149-3p	136	28	0.21
mmu-miR-5112	168	40	0.24
mmu-miR-6538	169	44	0.26
mmu-miR-744-5p	63	18	0.29

**Table 2:** Upregulated exosome microRNAs 24 h after rIPC.

microRNAs	sham	rIPC	Fold change
mmu-miR-875-5p	16	59	3.62
mmu-miR-98-3p	38	131	3.44
mmu-miR-126-3p	20	67	3.28
mmu-miR-670-5p	19	60	3.20
mmu-miR-411-5p	19	56	2.92
mmu-miR-344-5p	20	58	2.87
mmu-miR-3058-3p	17	44	2.62
mmu-miR-1249-5p	20	50	2.50
mmu-miR-686	19	48	2.47
mmu-miR-146a-5p	18	44	2.46
mmu-miR-488-5p	16	39	2.43
mmu-miR-3073a-5p	19	42	2.16
mmu-miR-7a-2-3p	18	39	2.16
mmu-miR-3961	20	43	2.13
mmu-miR-33-3p	17	35	2.11
mmu-miR-30b-3p	17	35	2.08
mmu-miR-652-3p	18	36	2.06
mmu-miR-125b-1-3p	16	33	2.01
