# Supplementary material for: Increased plasma VEGF levels following ischemic preconditioning are associated with downregulation of miRNA-762 and miR-3072-5p
Source: Sci Rep. 2016 Dec 1;6:36758. doi: 10.1038/srep36758 (PMC5131337; doi:10.1038/srep36758)
Supplement: Supplementary Information [file srep36758-s1.pdf]

Supporting online figure for:

Increased plasma VEGF levels following ischemic preconditioning are associated with downregulation of miRNA-762 and miR-3072-5p

Koji Ueno, Makoto Samura, Tamami Nakamura, Yuya Tanaka,Yuriko Takeuchi, Daichi Kawamura, Masaya Takahashi, Tohru Hosoyama, Noriyasu Morikage, Kimikazu Hamano

To whom correspondence should be addressed to:
Koji Ueno (E-mail: kjueno@yamaguchi-u.ac.jp)

This PDF file includes Supplementary Figure S1.

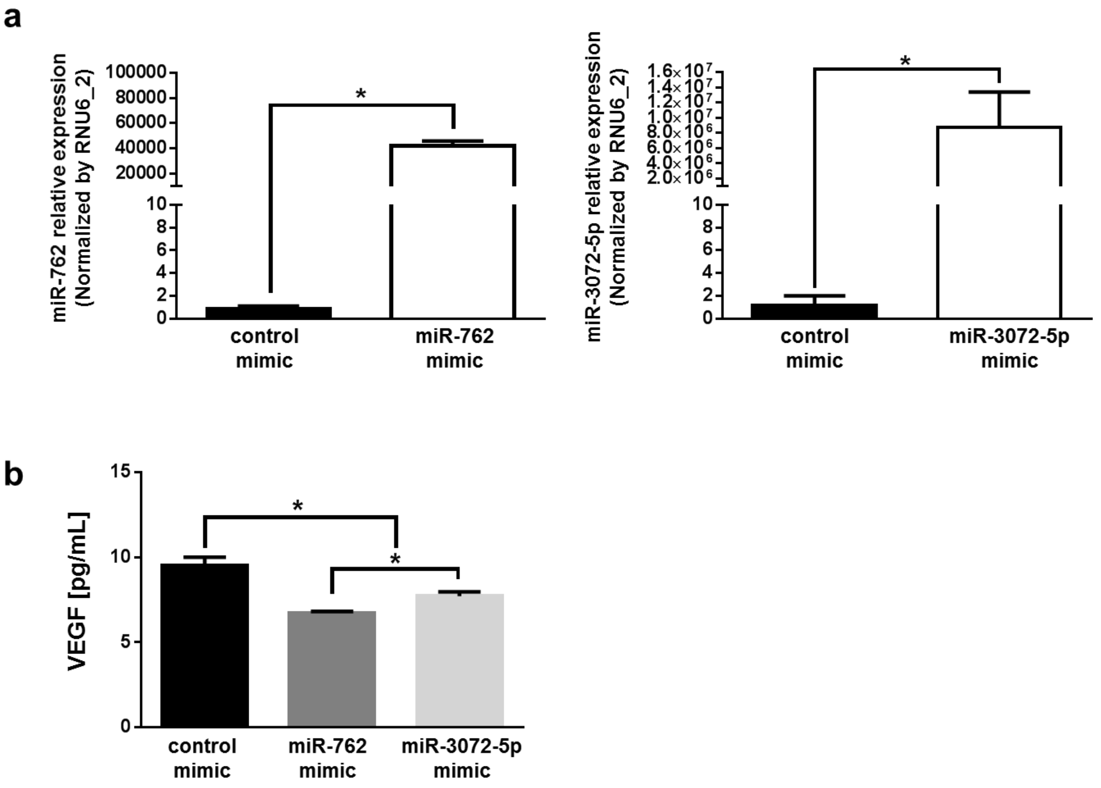

Supplementary Figure S1.

miR-762 and miR-3072-5p mimics inhibit VEGF production in CD34-positive BM cells

(a) Overexpression of microRNA expression after transfection with miR-762 and miR-3072-5p mimics. MicroRNA was extracted 24 h after transfection, and microRNA expression levels were confirmed using qPCR with RNU6_2 as an endogenous control. Expression levels were presented relative to those in CD34-positive BM cells that were transfected with control mimic. (b) VEGF secretion in CD34-positive BM cells 24 h after transfection. MicroRNA mimics were transfected into CD34-positive BM cells, and VEGF concentrations in media were measured using ELISA 24 h after transfection.
